# Enhancement of silicon modulating properties in the THz range by YAG-Ce coating

**DOI:** 10.1038/s41598-020-63386-w

**Published:** 2020-04-20

**Authors:** Jiu-sheng Li, Mu-shu Hu

**Affiliations:** Centre for THz Research, China Jiliang University, Hangzhou, 310018 China

**Keywords:** Organic-inorganic nanostructures, Two-dimensional materials

## Abstract

Y_3_Al_5-x_Ga_x_O_12_:Ce^3+^,V^3+^ (YAG:Ce) has excellent chemical stability and unprecedented luminous efficiency. Its strong photoresponsive property is thoroughly utilized in designing excellent optical information storage device. Here, the remarkable photoconductivity of YAG:Ce is exploited to demonstrate a hybrid YAG:Ce-silicon device that shows high speed terahertz wave spatial modulation. A wide terahertz spectra modulation is observed under different pump powers in frequency range from 0.2 to 1.8 THz. Furthermore, a dynamic control of the terahertz wave intensity is also observed in the transmission system. The modulation speed and depth of the device is measured to be 4 MHz (vs 0.2 kHz)and 83.8%(vs50%) for bare silicon, respectively. The terahertz transmission spectra exhibits highly efficiency terahertz modulation by optically pumping a YAG:Ce film on silicon with low optical pump fluence.

## Introduction

Terahertz wave functional devices are widely used in terahertz wireless communication, sensing and imaging systems^1–3^. These terahertz devices mainly include terahertz filters^4,5^, polarizers^6,7^, power dividers^8,9^, modulators^10–12^, absorbers^13,14^ and switches^15,16^, etc. But, in terahertz wireless communication system, terahertz wave modulator is the real core functional device. Over the past decades, several techniques have been reported to implement active terahertz modulators such as photonic crystals^17^, phase change materials^18^, organic materials^19,20^, liquid crystals^21–23^, graphene and two-dimensional (2D) materials^24–26^, etc. However, these modulators can not achieve broadband frequency range, high modulation speed and modulation depth at the same time. Thus, it is very meaningful to introduce new materials into terahertz regime for overcoming this terahertz wave modulation problem.

In this work, we demonstrate a high speed and photoactive terahertz wave radiation spatial light modulator using the photoinduced conductivity change ofYAG:Ce/Si hybrid structure. Spin-coating the solution processed YAG:Ce on silicon offers unprecedented advantages over the current silicon materials^27^ used in the fabrication of active terahertz modulator in terms of the cost, highly efficient modulation and simplification. We have measured an amplitude modulation of the terahertz transmission in the frequency range from 0.2 to 1.8THz with various laser intensity irradiances. The photoexcitation of YAG:Ce layer spin coated on the silicon actively modulates the terahertz wave with a modulation depth of 83.8% and modulation speed of 4MHz under the low laser pumping power of 0.2W/cm^2^. As photon-generated carriers in YAG:Ce/Si interface offer a strong modulation of the terahertz wave, such YAG:Ce/Si hybrid structure can aid in enhancing the terahertz wave energy manipulation efficiencies and could play a significant role in realizing high speed and broadband modulation with integration in terahertz silicon chip.

### Sample preparation and test

**Figure 1:**
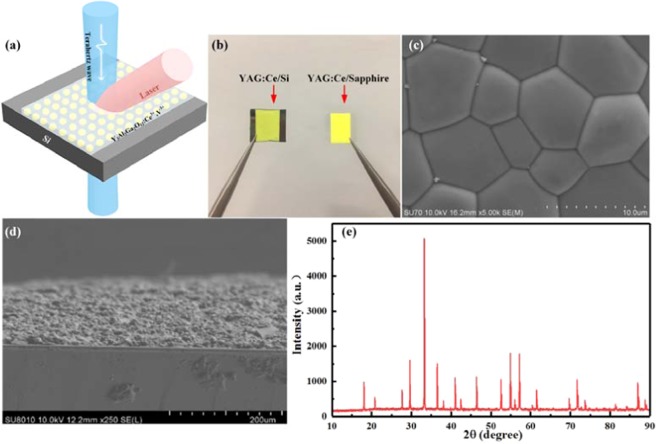
(**a**) Illustration of YAG:Ce/Si sample and experimental configuration, (**b**) Photography of YAG:Ce/Si and YAG:Ce/sapphire samples, (**c**) Surface SEM images of YAG:Ce sample, (**d**) Cross sections of YAG:Ce-on-silicon SEM image, (**e**) XRD patterns of YAG:Ce sample.

The YAG:Ce/Si hybrid structure terahertz modulator architecture and photography are revealed in Fig. 1(a,b). The synthesized phosphors were crushed into fine powers. The phosphors were blent with organic vehicles in a weight ratio to form viscous phosphor slurries. The phosphor slurries were carefully flowed on a 500µm-thick high-resistivity (>10000Ωcm) silicon substrate. After heating, remelting and natural cooling, the mixed film structure was finally obtained. Figure 1(c,d) depict the surface SEM images and cross-sectional scanning electron microscopy (SEM) image of the sample, respectively. To further characterize Y_3_Al_5-x_Ga_x_O_12_:Ce^3+^,V^3+^film, the X-ray diffraction (XRD) pattern of YAG:Cesample is measuredby using an X-ray diffractometer, as illustrated in Fig. 1(e).

**Figure 2:**
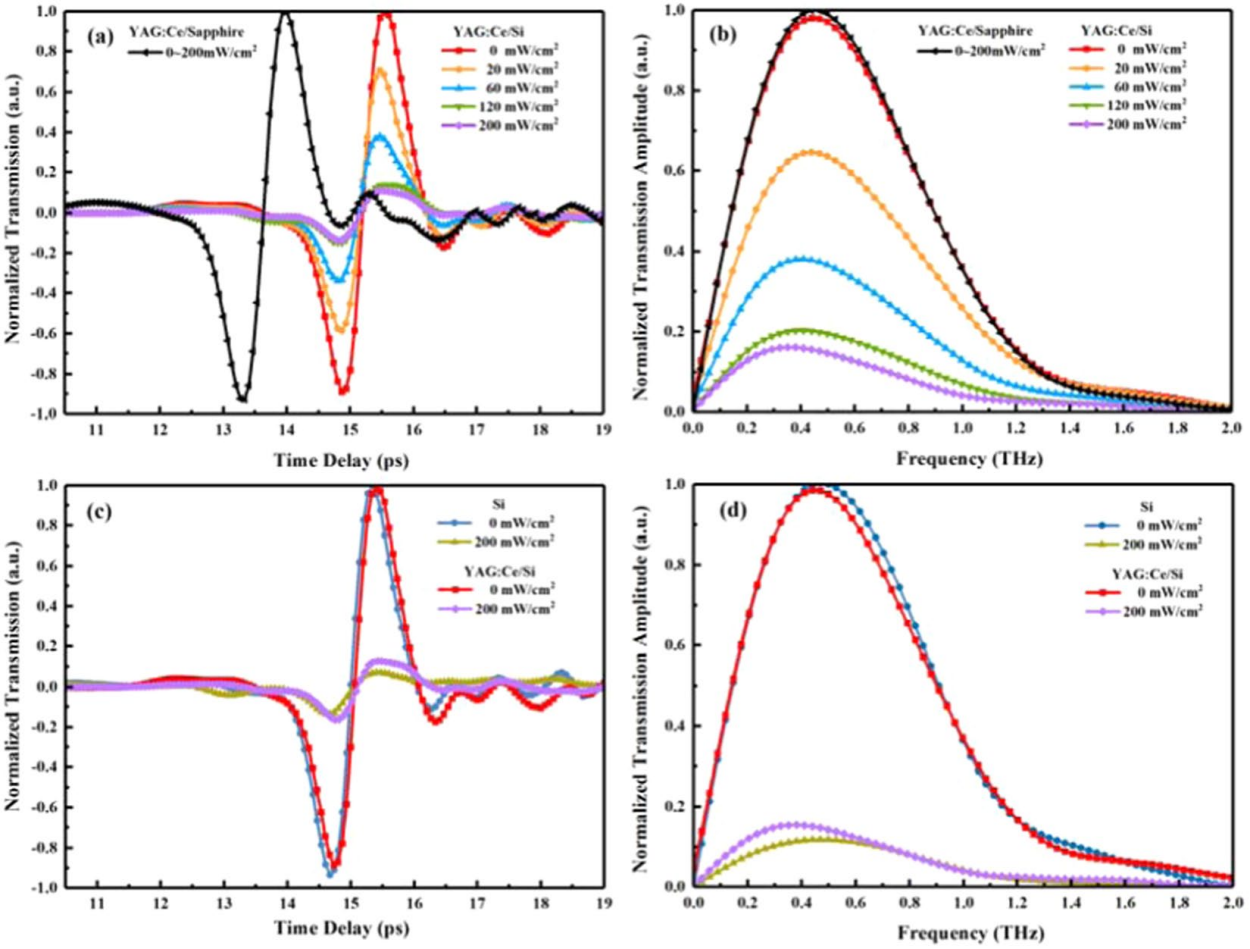
(**a**) and (**c**) Measured terahertz time-domain signals of the YAG:Ce/Si structure and bare silicon, (**b**) and (**d**) transmission spectra of the YAG:Ce/Si structure and bare silicon for varying powers of the optical photoexcitation pulse extracted using optical pump terahertz probe measurements.

Optical characterization of the YAG:Ce/Si sample was performed using the optical pump terahertz probe measurements in a LT-GaAs photoconductive antenna based terahertz time-domain spectroscopy (THz-TDS) system. The excitation source of THz-TDS was a Ti:sapphire laser with 100fs duration at 82MHz repetition rate, working at the wavelength of 800 nm. A ZnTe nonlinear crystal was used to detect the terahertz signal. The samples were placed at the confocal position of the system. The recorded terahertz time-domain signals are shown in Fig. 2(a,c), for varying pump fluences of the optical excitation pulse. In this experiment, a 800 nm continuous wave laser was used to irradiate the YAG:Ce film. The 800 nm pump laser plays a key role in producing photoinduced carriers in the YAG:Ce/Si interface. The terahertz wave spot is overlapped by the 800 nm continuous wave laser spot. For YAG:Ce/sapphire structure, one can see that there is no change in terahertz time-domain spectra under different intensities of laser irradiation. But for YAG:Ce structure, the main pulse amplitude of the terahertz time-domain spectra is a function of the pump laser power. The terahertz transmission for the proposed structure decreases with the increase of the pump laser fluences. Figure 2(b,d) show the transmission spectra for the YAG:Ce/Si structure and bare silicon for varying powers of the optical photoexcitation pulse extracted using optical pump terahertz probe measurements. The terahertz transmission spectrum of the YAG:Ce/Si hybrid structure and bare silicon has a significant decrease with the increase of the pump laser intensity.

### Experimental results and analysis

**Figure 3:**
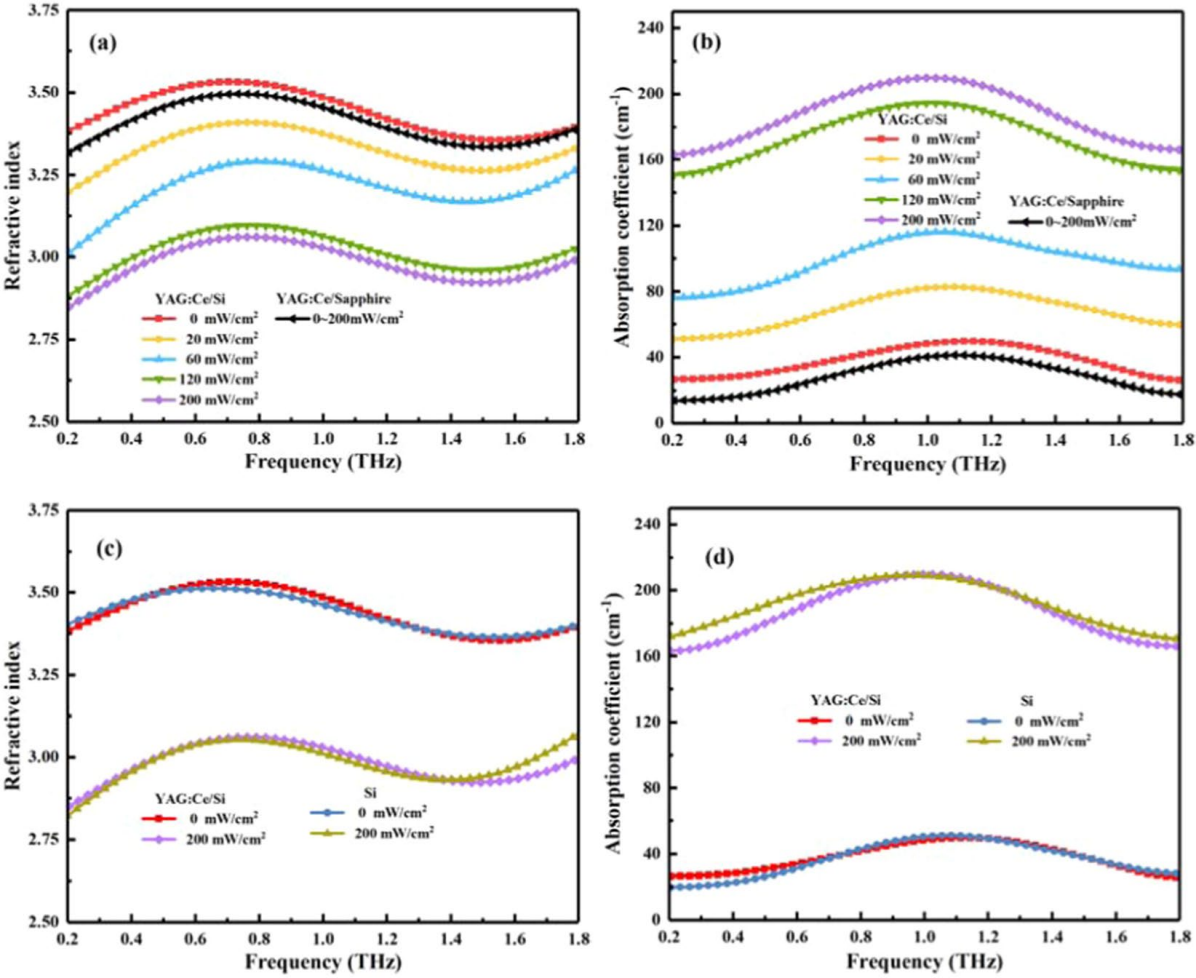
Frequency-dependent dielectric constants of the sample and bare silicon under different pumping power (**a**) refractive index, (**b**) absorption coefficient.

**Figure 4:**
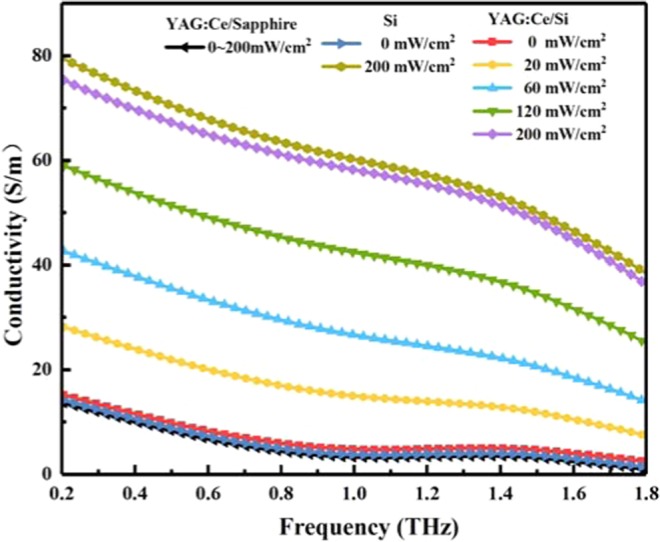
Frequency-dependent conductivity of the sample under different pumping power.

The present work is an experimental demonstration of Y_3_Al_5-x_Ga_x_O_12_:Ce^3+^,V^3+^-based active control of terahertz wave in a hybridYAG:Ce/Si structure system, wherein the photoexcitation of YAG:Ce layer spin coated on the silicon actively modulates the terahertz wave. To investigate the modulation mechanism, we deduced the optical constants of the YAG:Ce/Si devices via THz-TDS under various laser irradiances, as shown in Fig. 3. The refractive index and extinction coefficient can be defined as *n*(*ω*)=*c*·ϕ(*ω*)/(*ωd*) + 1 and *κ*(*ω*) = −*c*·ln{A(*ω*)·[*n*(*ω*) + 1]^2^/4*n*(*ω*)}/(*ωd*) from the time-frequency spectrogram^28,29^, respectively. And then, the absorption coefficient can be described as *α*(*ω*) = 2*ω*·*κ*(*ω*)/c^20,21^, where *A*(ω) and ϕ(ω) are the amplitude and phase of the transmission coefficient of the sample, respectively, *c* is the speed of light and *d* is the thickness of the sample. From Fig. 3, one can notice that the refractive index drops from 3.41 to 2.88 and the absorption coefficient increases from 26.53 cm^−1^ to 164.15 cm^−1^ for YAG:Ce/Si hybrid structure at 0.27THz as the laser pump intensity increases form 0W/cm^2^ to 0.2W/cm^2^. While both the refractive index and the absorption coefficient of the YAG:Ce/sapphire sample do not change under different pumping power. These phenomena are induced by the variation on conductivity, which is related to the carrier density. Meanwhile, the complex dielectric constant of the proposed structure can be described by *ɛ*(*ω*) = *ɛ*_*real*_(*ω*) + *iɛ*_*imag*_(*ω*), where *ɛ*_*real*_(*ω*) = [*n*(*ω*)]^2^ − [*κ*(*ω*)]^2^, *ɛ*_*imag*_(*ω*) = 2*n*(*ω*)*κ*(*ω*). Based on this, the conductivity *σ* can be given by *σ*(*ω*) = *ωɛ*_0_*ɛ*_*imag*_(*ω*). Figure 4 shows the conductivity data came from the experiment data of the sample. As the pumping power increases from 0 to 0.2W/cm^2^, the conductivity σ of the YAG:Ce/Si sample changes from 14.0S/m to 73.53S/m at 0.27THz. For comparison, we also performed numerical calculations the conductivity of the YAG:Ce/sapphire and bare silicon sample using the experiment data, as shown in Fig. 4. When the pumping laser illuminates the YAG:Ce/sapphire under the same pumping condition as the YAG:Ce/Si sample, it has no effect on the refractive index, absorption coefficient and conductivity of the YAG:Ce/sapphire as laser pumping intensity increase. It is well known that the sapphire is a kind of insulator and free carriers can not be generated under external applied photoexcitation. YAG:Ce film (about 30µm) is too thin to excite enough free carriers by itself under continuous wave laser pumping. To further support our claims on terahertz wave modulation using the YAG:Ce/Si hybrid structure, we performed numerical calculations using the Drude model^30,31^. The conductivity of YAG:Ce/Si hybrid structure is given by *σ=ne*^2^/[m^*^(1/*τ*-i*ω*)], where *n* is the carrier density which is determined by laser pumping power, *e* is the electron charge, m^*^ is the effective mass of the carrier, *τ* is the averaging relaxation time of the carrier, and ω is the circular frequency. According to the Drude model, one can obtain that when the laser pumping illumination power increases from 0 to 0.2W/cm^2^, the density of the photoexcitation carriers is about 7 times as much as that of without laser pumping. A large number of photocarriers increase the absorption of terahertz wave transmission, which will be beneficial to achieve broadband terahertz wave modulation and large modulation depth demonstrated by our experimental results.

**Figure 5:**
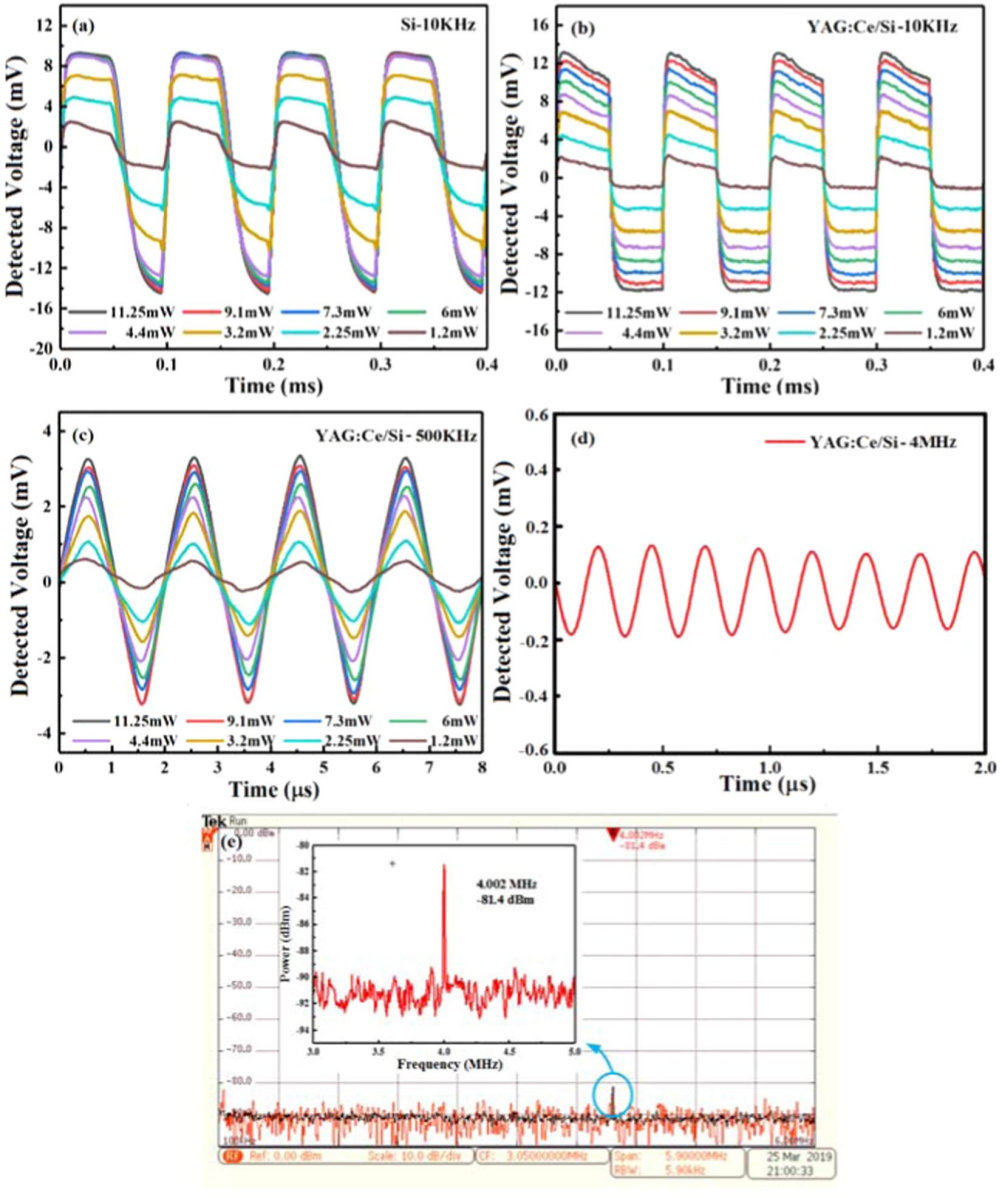
Experimental dynamic modulation (A 340 GHz Virginia Diodes CW terahertz source andzero-bias Schottky diode intensity detector is used for dynamic modulation measurements.) of the bare silicon (**a**) 10 KHz, and the YAG:Ce/Si sample under different modulated laser irradiation at (**b**) 10 KHz, (c) 500 KHz, and (**c–e**) 4 MHz under laser power with 11.25 mW (Here, (**e**) shows the detected energy spectra obtained by Tektronix signal, spectrum, and modulation analyzers).

**Figure 6:**
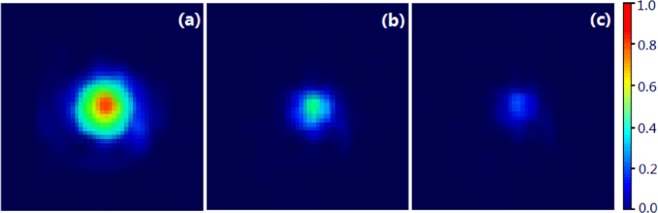
Terahertz wave intensity distributions through the YAG:Ce/Si hybrid structure under different laser radiation intensities of (**a**) 0 mW∕cm^2^, (**b**) 0.02W∕cm^2^, and (**c**) 0.2W∕cm^2^.

The fabricated Y_3_Al_5-x_Ga_x_O_12_:Ce^3+^,V^3+^/Si sample was also used to examine the performance of the dynamic modulation of continuous wave terahertz wave at frequency of 0.27THz. The modulated laser produces square laser pulses with 50% duty cycle. Figure 5 plots the measured dynamic characteristics of the proposed YAG:Ce/Si hybrid structure with various laser pump fluences. It also confirms that the photo-excitation free carriers give rise to the attenuation of the terahertz wave transmission. Figure 5(a,d) ~ illustrate the detected voltage signals waveform shape for modulation speed of 10KHz, 500KHz, and 4MHz. One can notice that the transmission amplitude of terahertz wave decreases with the modulation speed increase from 10KHz to 4MHz. For the modulation speed of 10KHz, the detected terahertz signal is square wave voltage. As the modulation speed increases to 500KHz, the modulated terahertz waveform becomes a triangle wave voltage. At modulation speed of 4MHz, the detected modulation voltage falls to 0.2 mV. Here, the modulation factor is defined as *MD*=1 − ∫*T*_laser-on_(*ω*)/∫*T*_laser-off_(*ω*)^23^, where *T*_laser-on_ and *T*_laser-off_ are the transmitted terahertz powers without and with laser radiation, respectively. In order to visually observe the effect of different laser irradiation intensity on terahertz transmission, we employed a terahertz camera system to obtain the terahertz transmission intensity image. When the pumping laser irradiates the silicon, the electrons gain photon energy through intrinsic absorption and then undergo excited transitions, resulting in photoinduced electron hole pairs. At this point, the conductivity of the sample increases with the increase of non-equilibrium carrier concentration. Under the condition that the irradiation intensity of the pumped laser remains unchanged, the conductivity increases with the increase of irradiation time due to the accumulation of carrier concentration, which leads to the increase of modulation depth. Since the photoexcitation is accompanied by the recombination of non-equilibrium carrier pairs, the photogenic carrier concentration will eventually stabilize with time and the modulation depth will appear saturation phenomenon. When the pumping laser is not applied, the photoconductivity of the silicon material decreases with the carrier recombination process. Because the intrinsic silicon lattice is complete and free of impurities, its modulation rate is limited by its carrier lifetime, so the modulation rate of the bare silicon is low [The modulation speed of the bare silicon is 0.2 kb/s. see ref. ^32^. Due to the special energy gap (valence band, conduction band position) between the two materials (YAG:Ce and Si), the interface is in the potential well of energy band. The carriersare constrained to move in a very small space. This special interface forms a two-dimensional electron gas.Because of being confined to a small space, the migration rate of the carriers in this space will be greatly increased, which affects the response speed of the device. Therefore, the rise time and decay time of YAG:Ce/si structure are faster than those of the bare silicon, and the modulation rate is significantly improved. Figure 6 shows the terahertz wave transmission intensity distribution through the YAG:Ce/Si hybrid structure without and with laser pumping. According to the Figure, it is worth to point out that the terahertz transmission intensity is dependent on the external laser pumping power. Figure 6(a) illustrates the terahertz transmission intensity distribution through the YAG:Ce/Si structure without laser pumping power. Under 0.2W/cm^2^ laser pumping intensity, the terahertz transmission intensity dropped to 17.2% of its original value, as shown in Fig. 6(c).

**Figure 7:**
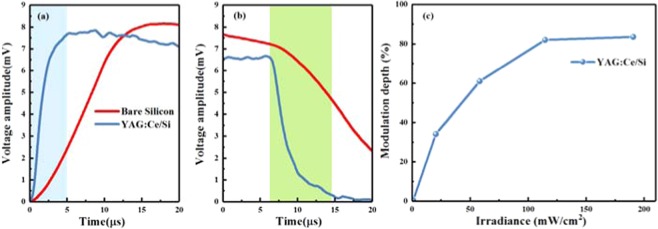
Rise time (**a**), decay time (**b**), and modulation depth (**c**) under different laser pumping power.

The modulation speed and depth are two key parameters of the terahertz wave modulator. Figure 7(a,b) show the response speed of the proposed device. From the figure, one can see that the rise time and decay time of YAG:Ce/Si structure sample are much faster than those of the bare silicon. The sample reaches saturation within 5µs, while bare silicon is beyond 15µs. The results confirm that the proposed structure can provide a modulation speed of 4MHz. As shown in the Fig. 7(c), the modulation depth of the device increases at very low laser irradiation power (~0.02W/cm^2^) and saturates as the laser irradiation intensity at 0.2W/cm^2^. Then, we get the corresponding modulation depth of 83.8%. The experimental results show that the designed terahertz modulator has the advantages of simple structure, high modulation speed, high modulation efficiency, easy manufacturing, and has promising application prospects in terahertz wireless communication, sensing and imaging systems.

## Conclusion

In summary, we have experimentally demonstrated that YAG:Ce is an ideal dynamic material for increasing terahertz wave modulation speed. A broadband modulation of the terahertz transmission in the frequency range from 0.2 to 1.8 THz isobtained. Dynamic experiments at 0.27 THz carrier evidence that the YAG: Ce/Si hybrid structure achieves the modulation depth of 83.8% (vs 50% for bare silicon) and the modulation speed of 4 MHz (vs 0.2 kHz for bare silicon) under an external pumping laser power of 0.2 W∕cm^2^. We performed numerical calculations to validate our experimental results. The proposed YAG: Ce/Si terahertz wave modulator can be easily integrated in silicon substrate chip and has a promising application in terahertz science and technology.

## Methods

We have characterized using typical terahertz time-domain spectroscopy (THz-TDS) system consisting ofa photoconductive antenna transmitter and a receiver. The measurement set-up for dynamic modulation characteristics consists of aVirginia Diodes CW terahertz source and Schottky diode intensity detector.
